# Unexpected Tolerance of α-Cleavage of the Prion Protein to Sequence Variations

**DOI:** 10.1371/journal.pone.0009107

**Published:** 2010-02-08

**Authors:** José B. Oliveira-Martins, Sei-ichi Yusa, Anna Maria Calella, Claire Bridel, Frank Baumann, Paolo Dametto, Adriano Aguzzi

**Affiliations:** Institute of Neuropathology, University Hospital of Zürich, Zurich, Switzerland; Universidade Federal do Rio de Janeiro (UFRJ), Brazil

## Abstract

The cellular form of the prion protein, PrP^C^, undergoes extensive proteolysis at the α site (109K↓H110). Expression of non-cleavable PrP^C^ mutants in transgenic mice correlates with neurotoxicity, suggesting that α-cleavage is important for PrP^C^ physiology. To gain insights into the mechanisms of α-cleavage, we generated a library of PrP^C^ mutants with mutations in the region neighbouring the α-cleavage site. The prevalence of C1, the carboxy adduct of α-cleavage, was determined for each mutant. In cell lines of disparate origin, C1 prevalence was unaffected by variations in charge and hydrophobicity of the region neighbouring the α-cleavage site, and by substitutions of the residues in the palindrome that flanks this site. Instead, α-cleavage was size-dependently impaired by deletions within the domain 106–119. Almost no cleavage was observed upon full deletion of this domain. These results suggest that α-cleavage is executed by an α-PrPase whose activity, despite surprisingly limited sequence specificity, is dependent on the size of the central region of PrP^C^.

## Introduction

The prion protein is necessary for the development of prion diseases, also termed transmissible spongiform encephalopathies (TSEs) [1], [2]. The most established molecular model for these diseases proposes that the cellular form of the prion protein (PrP^C^) is refolded into β-sheet-rich aggregates (PrP^Sc^), which constitute the infectious agent termed prion [3], [4]. While the concept of a nucleic-acid-free infectious agent spreading through protein aggregation is supported by a large body of evidence, the mechanisms by which PrP^Sc^ aggregates and induces neurotoxicity are poorly understood [4], [5].

Studies on proteolytic processing of PrP^C^ and PrP^Sc^ have shown that PrP^C^ is mainly cleaved in a region termed the α-cleavage site [6]. In human, bovine, and ovine PrP^C^, the cleavage has been reported to occur at the conserved residues 109K↓H1110 [7], [8], [9] (the residue numbering of murine PrP^C^ is used in this report) in the unstructured portion of PrP^C^ [10]. The resultant products are the globular C-proximal and the flexible N-proximal domains (C1 and N1, respectively) [7]. In contrast, the disease-associated PrP^Sc^ conformer undergoes a process named β-cleavage [6], which generates a larger C-proximal product designated C2 [7], [11]. One likely explanation for this differential processing is that the α-cleavage site may be buried within the aggregates of PrP^Sc^ [7], [11] and may therefore be protected from proteolysis.

The subcellular site of α-cleavage is controversially discussed. On the one hand, cleavage is inhibited by ammonium chloride, suggesting that it occurs in an acidic endocytic compartment [12]. On the other hand, C1 accumulates intracellularly in human SH-SY5Y cells expressing secreted anchorless PrP^C^, suggesting that cleavage occurs in a late compartment of the secretory pathway [13].

Certain PrP^C^ deletion mutants, such those termed ΔF (PrP^Δ23–134^) and ΔE (PrP^Δ23–121^) [14], resemble the C1 cleavage product [7], [14] and do not support prion replication [5], [15]. Therefore, enhancement of α-cleavage *in vivo* may diminish the availability of full-length PrP^C^ for conversion into PrP^Sc^. If this conjecture proves correct, it will be important to identify the proteases responsible for cleavage of PrP^C^, here termed α-PrPases, and any hypothetical factors that may control their activity.

Moreover, certain PrP^C^ variants bearing deletions close to the α-cleavage site induce toxicity when transgenically expressed in mice. Toxicity can be rescued in a dose-dependent manner by coexpression of normal PrP^C^ [14], [15], [16], [17]. This raises the question whether the toxicity of these PrP^C^ mutants may be related to abnormalities in the proteolytic processing of PrP^C^.

But which structural determinants of PrP^C^ control the interaction with the putative α-PrPase? When expressed in transgenic mice, PrP^C^ mutants bearing large deletions of the α-cleavage site, such as PrP^Δ94–134^, had a complete cleavage inhibition and were highly toxic in *Prnp* ^−/−^ mice [14], [16], whereas the downstream deletion PrP^Δ114–121^ exhibited some cleavage and showed only mild toxicity [14]. This *in vivo* data on proteolytic processing of PrP^Δ94–134^ replicate the observation that PrP^Δ95–132^ is not cleaved when expressed in Hpl cells [18].

Other studies on PrP^C^ processing in cultured cells suggest that the alanine and glycine residues in the PrP^C^ palindromic region localized in the downstream flank of the α-cleavage site may be important for α-cleavage [19]. It was reported that in transfected N2a cells, PrP^C^ bearing two point mutations that transformed the palindromic domain 112–119 into an alanine chain had no remarkable impairment in α-cleavage [19]. Conversely, the cleavage product C1 was not, or very weakly, detectable in constructs where this palindrome was converted into a glycine chain, or where region 105–125 was deleted [19]. Other studies also suggested that α-cleavage is not affected by the removal of the octapeptide region [15], [18], [20] or by the insertion of supernumerary octapeptide repeats [20]. α-cleavage of PrP^C^ mutants was also assessed in cells expressing ovine PrP (ovPrP) with GFP inserted into the N-proximal region [21]. In this system, the point mutations ovPrP^K113R^, ovPrP^K113D^, ovPrP^K113A^ and ovPrP^KHV113–115AAA^ did not appear to affect cleavage, with the respective mutant proteins processed similarly to the fusion protein of wild-type ovPrP^C^ with GFP.

Here we attempted to test certain predictions regarding the sequence requirements to PrP^C^ for α-cleavage. We confirmed that the murine PrP^C^ domain 106–119 is indeed important for PrP^C^ cleavage. In addition, we found that PrP^C^ cleavage was independent of the precise sequence at the cleavage site, and was largely independent of the charges and hydrophobicity in the vicinity of α-cleavage site. Instead, the efficacy of α-cleavage was linearly dependent on the size of deletions between residues 106–119. Likewise, substitutions of alanines to glycine residues in the PrP^C^ palindromic region 112–119 had no influence on the degree of C1 generation. Finally, we did not detect any C1 generation in lysates of murine *Prnp^−/−^* cells and brains expressing anchorless secreted PrP^C^ (secPrP^C^) indicating that cleavage is membrane anchor-dependent.

## Results

### Characterization of α-Cleavage of PrP^C^ in Various Cell Lines

**Figure 1:**
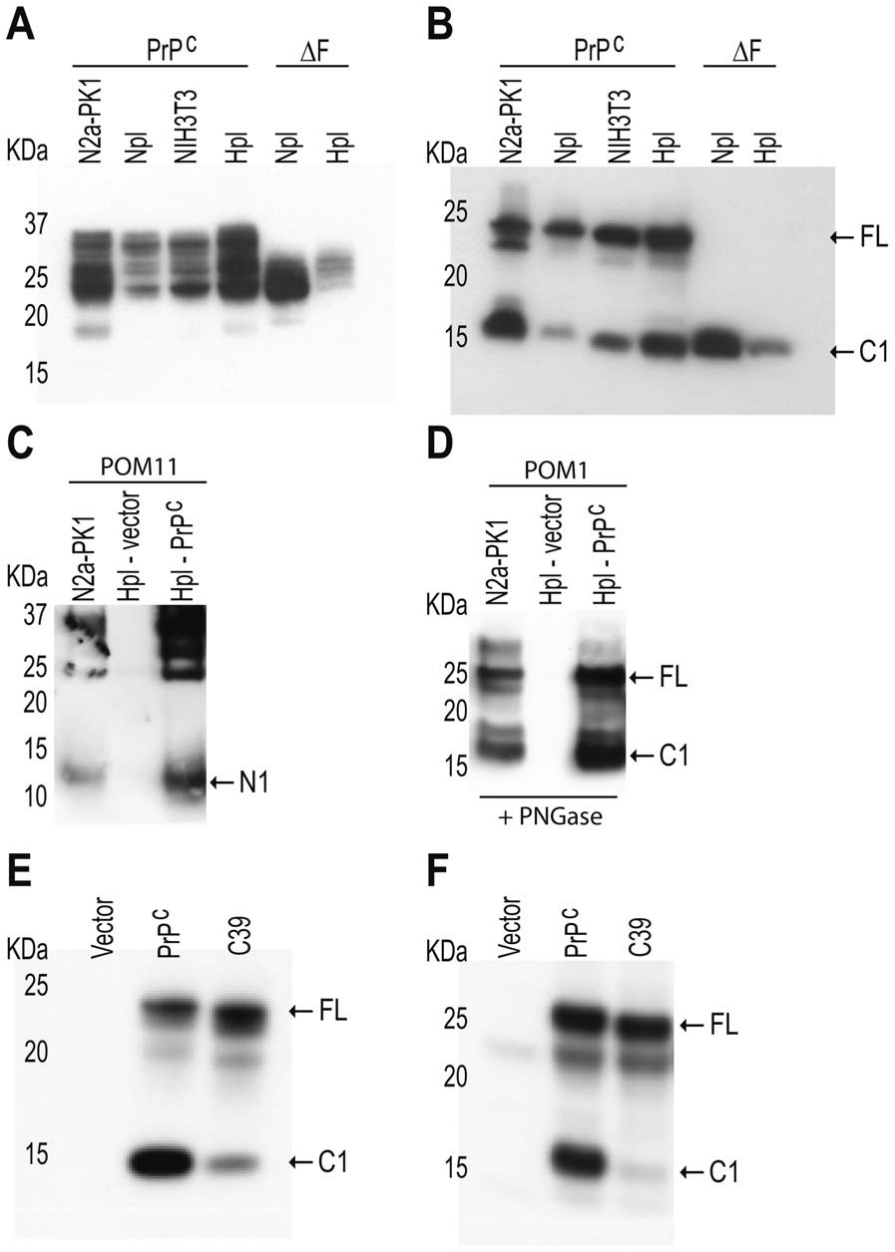
Proteolysis of PrP^C^ in various biological models. **A–B**: Western blots of lysates of various cell lines (N2a-PK1, Npl, NIH3T3, Hpl) non-treated (A) or treated (B) with PNGase. Cells were transfected with a plasmid encoding PrP^C^ or PrP^Δ32–134^; (lanes labeled PrP^C^ and ΔF, respectively). Detection was performed with antibody POM1 which recognizes the globular domain of PrP. **C–D**: Western blot of cell culture media (C) or cell lysates (D) from N2a-PK1 cells and PrP^C^-transfected or parental Hpl cells (lanes labelled accordingly). Media (C) were immunoprecipitated with antibody POM2 and detected with POM11, which both recognize the N-proximal region of PrP^C^, whereas cell lysates (D) were treated with PNGase and detected with POM1. **E**: Western blot of PNGase-treated lysates of human HeLa cells transfected with murine PrP^C^ or with construct C39 (PrP^Δ111–120^). Detection was performed with antibody POM19 which recognizes the C-proximal domain of murine, but not human, PrP^C^. **F**: Western blot of PNGase-treated cell lysates of mouse primary mouse embryonic fibroblasts (MEFs) from *Prnp^−/−^* mice. MEFs were transfected with constructs encoding murine PrP^C^ or C39 (PrP^Δ111–120^). Arrows point to the full length PrP (FL) and to the C1 fragment.

We assessed the glycosylation and α-cleavage of PrP^C^ in cell lines transfected with constructs encoding PrP^C^ and the mutant ΔF [15], which almost completely lacks the N-proximal region and displays an electrophoretic mobility similar to that of C1 [14], [15] (Fig. 1A–B). The cell lines used were the murine neuroblastoma cell line N2a-PK1 [22], the murine fibroblast cell line NIH3T3 [23], and the Npl and Hpl3–4 cell lines derived from *Prnp*-ablated mice [24], [25].

We found that the extent of glycosylation of both PrP^C^ and ΔF was quantitatively and qualitatively similar in each of the above cell lines (Fig. 1A), and essentially corresponded to that of brain tissue (data not shown). Upon deglycosylation with PNGase, a sharp C1 band with electrophoretic mobility similar to that of ΔF (ca. 15 kDa) was detectable in all cell lines (Fig. 1B). The N-proximal fragment of α-cleavage was subsequently assessed in N2a-PK1 cells and in PrP^C^-transfected Hpl cells. PrP^C^ was immunoprecipitated from culture media with antibody POM2 and detected with antibody POM11. Both POM2 and POM11 react with the octapeptide repeat region of N-proximal PrP^C^ tail [26], and evidenced the N1 product of α-cleavage (ca. 12KDa; Fig. 1C).

The size of the N-proximal fragment observed here is in agreement with previous reports in murine TSM1 neurons and in human HEK293 cells [27], and with chicken PrP in murine N2a cells [28]. Instead, we did not confirm a report that the dominant N-terminal band in N2a and GT1 cells has an apparent molecular weight of 7.6 KDa [6]. The C1 fragment was also detected after PNGase treatment of total cell lysate of the same cultures (Fig. 1D). The observation of these two well-defined cleavage fragments, C1 and N1, supports the conjecture that α-cleavage is a controlled proteolytic process rather than mere exoproteolytic digestion of the unstructured region by nonspecific proteases.

**Figure 2:**
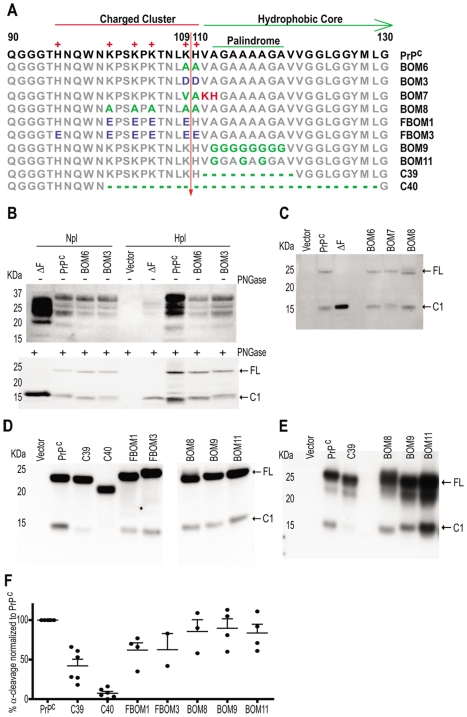
Proteolysis of PrP^C^ variants with mutations in the palindrome and in the positive charges neighbouring the α-cleavage site. **A**: Amino acid sequence alignment of the PrP^C^ constructs used. Numbers represent amino-acid residues in murine PrP^C^ sequence. The positive residues, the charged cluster, hydrophobic core and palindrome are indicated. Arrow: α-cleavage site. Grey: non-mutated residues. Green: deletions or mutations into non-charged residues. Blue: mutations into negatively charged residues. Red: mutations into positively charged residues **B**: Western blots of cell lysates of Hpl and Npl cells transfected with the various mutants. Samples in the lower blot were deglycosylated with PNGase. **C–D**: Western blot of PNGase-treated Hpl cells transfected with the various PrP^C^ mutants. All lanes in (D) belong to the same blot. Detection was done with POM1. **E**: Western blot of PNGase treated cell lysates of HeLa cells transfected with various PrP^C^ mutants. Detection was done with POM19. **F**: Quantification of the percentage of α-cleavage of the various PrP^C^ mutants, based on densitometry of western blots from which (D) is representative. Quantifications were calculated in the linear range of the densitometric signal of independent experiments. Values refer to the amount of C1 generation comparing to total abundance of PrP^C^, and are normalized to cleavage of wild-type PrP^C^, which was assessed in the same blot. Error bars represent standard errors of the mean (SEM). Arrows point to the full length PrP (FL) and to the C1 fragment, which can also correspond to ΔF.

To assess whether PrP^C^ is universally cleaved in other cell systems, PrP^C^ and the mutant C39 were transfected into human HeLa cells [29] (Fig. 1E) and into murine primary embryonic fibroblasts (MEFs) derived from *Prnp* ^−/−^ mice (Fig. 1F). C39 lacks the palindromic region that flanks the α-cleavage site of PrP^C^ (Fig. 2A), and a deletion within the same region was reported to impair C1 generation in transgenic mice [14]. In both HeLa and MEFs the murine PrP^C^ molecule was efficiently cleaved (Fig. 1E–F), whereas α-cleavage of the mutant C39 was impaired in both cell lines. We conclude that the structural features that control α-cleavage of PrP^C^ are common to many cell types of diverse histogenetic origin, including established murine cell lines, human cell lines, and murine primary cells.

### Impact of Residues Neighbouring the α-Cleavage Site onto PrP^C^ Processing

The α-cleavage site of PrP^C^ is located between a charge cluster (CC) and a hydrophobic core (HC), and is flanked N-proximally by positively charged residues and C-proximally by a palindrome sequence (Fig. 2A). We first investigated whether the positive charges at the α-cleavage site contribute to defining the proteolytic site. For this purpose we designed a series of PrP^C^ mutants with substitutions of the basic amino acids. In the BOM6 construct we mutated to neutral alanine residues the two charged amino acids of the α-cleavage site, whereas in BOM3 we mutated the positive charges into anionic aspartate residues (Fig. 2A). Because ΔF and C1 were previously found to display similar electrophoretic mobility, ΔF was used as a molecular size marker for identifying C1 [14], [15].

Cell lysates of lines transfected with PrP^C^ and its mutants were collected, and PrP^C^ glycosylation and processing were assessed before and after treatment with PNGase. BOM6, BOM3, and the deletion mutant ΔF all underwent glycosylation similarly to PrP^C^ in both cell lines. Therefore, charge inversion or large deletions in the flexible N-proximal domain of PrP^C^ did not exert any appreciable impact onto PrP^C^ glycosylation (Fig. 2B). This observation is in agreement with reports suggesting that large alterations in the region of the CC and HC have no measurable impact in the distribution of the mutants between various cellular microdomains [14], [30].

The N1 fragment is released into the medium, whereas C1 retains its GPI anchor and its membrane localization. Furthermore, N1 encompasses the unstructured domain of PrP [10] and is unstable. Because of these circumstances, we reasoned that the degree of cleavage of the various PrP^C^ mutants would be more reliably assessed by measuring C1 rather than N1. Therefore, we quantitated the C1 band and compared it to the integrals of the band corresponding to uncleaved PrP^C^ (Fig. 2B). In both Hpl and Npl cells the degree of α-cleavage of BOM6 and BOM3 was comparable to, or <25% lower than, that of wild-type PrP^C^ (Fig. 2B). Hence removal, or even inversion, of the positive charges at the α-cleavage site did not result in a remarkable inhibition of α-cleavage. The values for cleavage inhibition of the mutants varied <7% between Hpl and Npl cells (Fig. 2B), indicating that the pattern of proteolytic processing of PrP^C^ in these two cell lines was qualitatively and quantitatively similar. For this reason we focused on the Hpl cell line for most of the studies described in the following.

To further test the possibility that α-cleavage is independent of the charges and sequence of the cleavage site, we designed the BOM8 mutant in which all five basic histidine and lysine residues neighbouring the α-cleavage site were mutagenized into uncharged alanines (Fig. 2A). We also asked whether swapping the position of the α-cleavage site would alter the proteolytic processing of PrP^C^. For this the mutant BOM7 was elaborated, whose α-cleavage site was shifted downstream by two amino acids (Fig. 2A). We found that the cleavage efficiency of BOM6, BOM8 and BOM7 was between 95 and 100% of the cleavage of wild-type PrP^C^. Hence, as with the previously assessed constructs, BOM8 and BOM7 generated C1 with a similar efficiency to that of wild-type PrP^C^ (Fig. 2C).

Having established that the above modifications had little impact onto the α-cleavage, we assessed the impact of stronger alterations of the CC region. For this purpose we analyzed BOM8 together with two constructs bearing charge inversions. FBOM3 had all six cationic residues of the CC mutated into anionic glutamate residues, resulting in complete inversion of all charges (Fig. 2A). In FBOM1 the positive charges of four lysines were inverted into anionic glutamate residues, but these acidic residues were interspersed with two cationic histidines (Fig. 2A). Constructs C39 and C40 (which carries a large deletion of the region 100–129) were analyzed in parallel and used as “molecular milestones” for assessing moderate and extensive cleavage inhibition, respectively. Quantitation of the C1 fragment generated in these constructs and in wild-type PrP^C^ confirmed that removal of the five positive charges around the α-cleavage site and in the neighbouring region did not affect the proteolytic processing of PrP^C^ (Fig. 2D,F). Even extremely drastic modifications, such as the polarity inversion of four or even six positive charges (FBOM1 and FBOM3), resulted in only about 38% α-cleavage impairment (Fig. 2D,F). On the basis of these results it is unavoidable to conclude that the charges surrounding the α-cleavage site are largely irrelevant for the proteolysis of PrP^C^, with neutralization and even complete charge reversal of the CC region resulting in a surprisingly slight impairment of α-cleavage.

### Role of the PrP^C^ Palindromic Region (111–120) in α-Cleavage

Next, we studied the residues located carboxy proximally to the cleavage site. This region contains a palindrome which is immediately adjacent to the N-terminal boundary of the HC (Fig. 2A). A previous study reported that substitution of all palindromic glycines with alanines, as in construct BOM9, abrogated C1 generation in N2a cells [19]. We therefore assessed the cleavability of BOM9 and of BOM11, which bears only three palindromic alanine substitutions, in *Prnp^−/−^* Hpl cells [25] (Fig. 2A). To our surprise, quantification of C1 revealed that both constructs were processed by α-cleavage similarly to wild-type PrP^C^ (Fig. 2D,F). In order to evaluate the robustness of these results, the palindromic mutants BOM9 and BOM11, the palindromic-deletion construct C39, and the charge-neutralized BOM8 were transfected into HeLa cells. Fragments were analyzed with antibody POM19 which recognizes the globular domain of murine, but not of human, PrP^C^ [26]. The proteolytic processing of these mutants was indistinguishable in both HeLa (Fig. 2E) and Hpl cells (Fig. 2D,F) and was essentially identical to that of wild-type PrP^C^ (Fig. 2E). In contrast, C39 displayed partial α-cleavage inhibition in both Hpl and HeLa cells, which is in agreement with the finding that a mutant bearing a deletion of the region 114–121 is inefficiently cleaved in the brain of transgenic mice [14]. We conclude that, in contrast to published claims [19], the substitution of the palindrome with a total or partial polyglycine chain does not have a measurable impact on α-cleavage of PrP^C^.

### The Impact of Hydrophobicity onto α-Cleavage

Another striking feature that might modulate α-cleavage of PrP^C^ is the strong hydrophobicity of the residues neighbouring the cleavage site. Since the HC may be associated to the cell membrane [31], we reasoned that α-cleavage may be affected by the distance between the cleavage site and the cell membrane. In this scenario, reduction of the hydrophobicity of the HC may modify its interaction with the cell membrane and thereby decrease the rate of α-cleavage.

**Figure 3:**
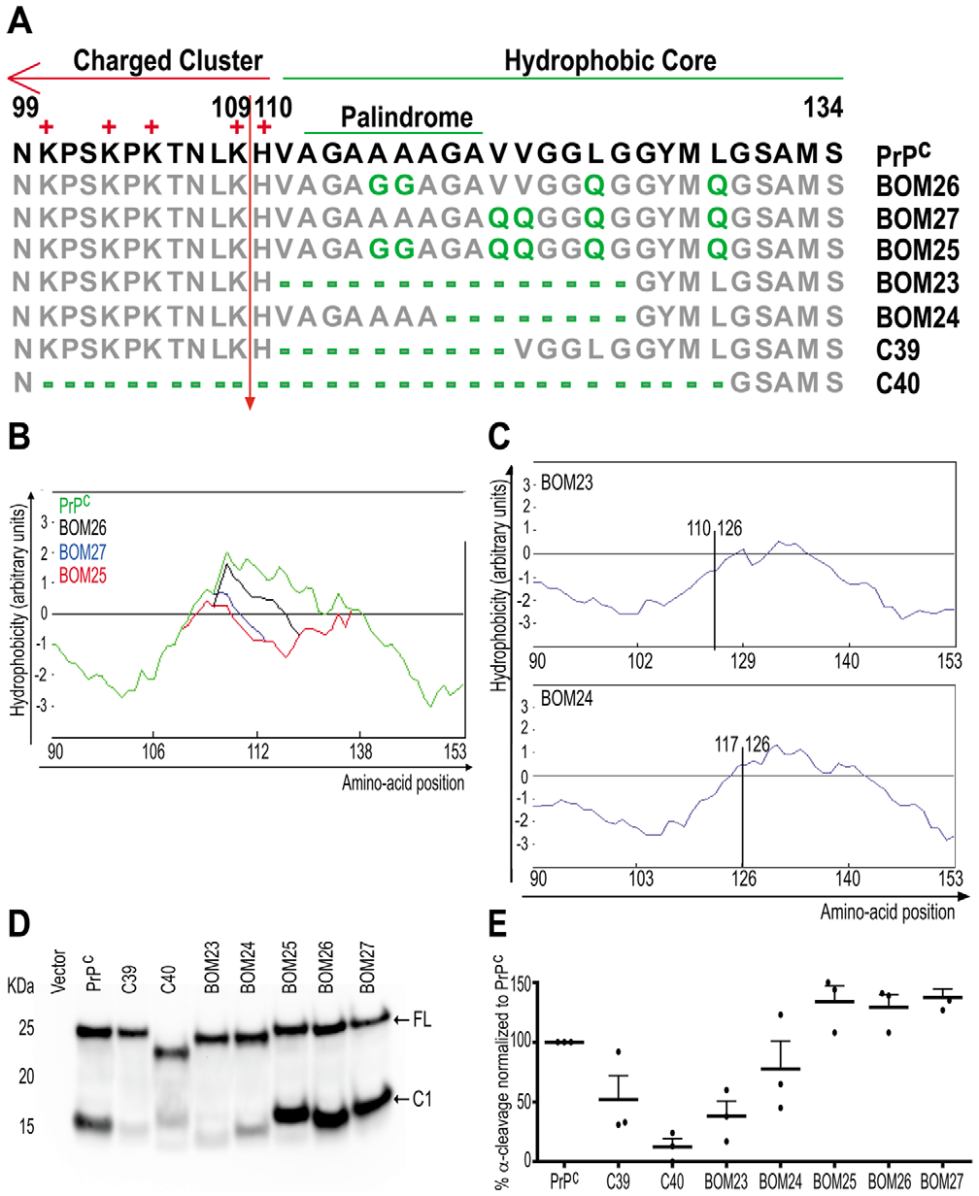
Evaluation of the role of the hydrophobicity in the α-cleavage site region in modulating α-cleavage. **A**: Amino acid sequence alignment of the PrP^C^ constructs used. Numbers represent amino-acid residues. The positive residues of the charge cluster, the hydrophobic core, and the palindrome are highlighted. Arrow: α-cleavage site. Grey: non-mutated residues. Green: deletions or mutations into non-charged residues. **B**: Superimposed hydrophobicity plots of the region 90–153 of PrP^C^ (green) and mutants BOM26 (black), BOM27 (blue), and BOM25 (red). **C**: Hydrophobicity plot of the region 90–153 of BOM23 in the upper panel and BOM24 in the lower panel. Vertical line represents the site of the deletion spanning the residues 111–125 and 118–125 for BOM23 and BOM24 respectively. Numbers adjacent to the vertical line indicate the amino acid residues flanking the deletion of the mutants. **D**: Western blot of PNGase treated cell lysates of Hpl cells transfected with the various PrP^C^ deletion constructs used in the current study. Detection was done with POM1. Arrows point to the full length PrP (FL) and C1 fragment. **E**: Quantification of the percentage of α-cleavage of the various PrP^C^ mutants, based on densitometry of western blots, from which (D) is representative. Quantifications were calculated in the linear range of the densitometric signal of independent experiments. Values refer to the amount of C1 generation comparing to total abundance of PrP^C^, and are normalized to cleavage of non-mutated PrP^C^, which was assessed in the same blot. Error bars represent the SEM.

To test the latter hypothesis, we designed three PrP^C^ constructs in which we substituted hydrophobic residues with non-ionic hydrophilic analogues of roughly similar molecular size. Accordingly, we substituted alanine with glycine residues, whereas leucines and valines were replaced with glutamines (Fig. 3A). The maximal deviation in primary sequence between each pair of constructs was four residues. Construct BOM26 displayed only a slight reduction of hydrophobicity and therefore was expected to be cleaved almost as efficiently as wild-type PrP^C^, whereas BOM27 had a stronger reduction of hydrophobicity (Fig. 3A–B). Finally, BOM25 bears almost no positive hydrophobic integral and was designed to stringently test whether hydrophobicity would play any role in α-cleavage (Fig. 3A–B). Again, constructs C39 and C40 were used in control experiments and served as molecular milestones of moderate and extensive inhibition of cleavage, respectively.

Quantification of C1 generation in constructs BOM26, BOM27, and BOM25 showed that none of these mutations impaired the proteolysis of PrP^C^ (Fig. 3D–E). We even observed slightly increased proteolysis of PrP^C^, but this increase was similar for all three mutants and was therefore independent of the hydrophobicity. Thus, the hydrophobicity of the region neighbouring the α-cleavage site appears to play no significant role in the proteolytic processing of PrP^C^.

We then took the more extreme approach of introducing large deletions into the HC. BOM24 had an eight-amino acid deletion which mildly reduced the hydrophobicity around the α-cleavage site (Fig. 3A,C), whereas BOM23 had a larger deletion extending upstream to the beginning of the HC and strongly reducing its hydrophobicity (Fig. 3A,C). Measurements of C1 (Fig. 3D–E) showed that α-cleavage was mildly inhibited in BOM24 and strongly inhibited in BOM23 (Fig. 3D–E). These results suggest that α-cleavage may be inhibited in mutants with low hydrophobicity in the HC or, alternatively, that inhibition is primarily dependent on the size of the deletion.

### Definition of a Domain Regulating α-Cleavage

**Figure 4:**
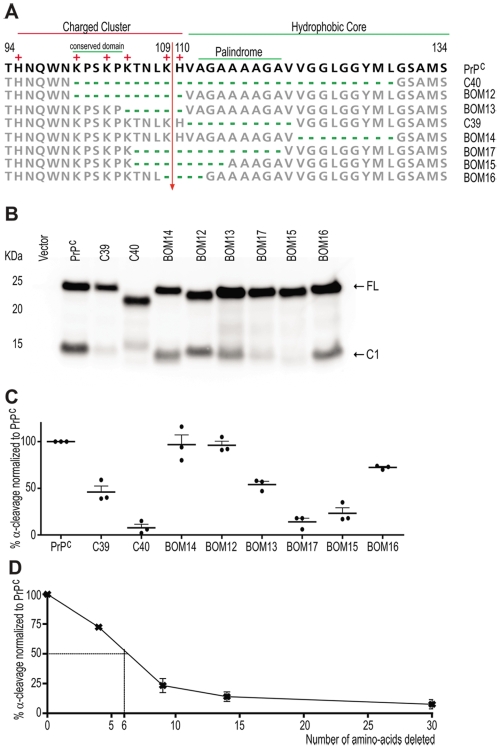
Search for a defined domain within PrP^C^ that regulates its α-cleavage. **A**: Amino acid sequence alignment of the PrP^C^ constructs used. Numbers represent amino acid residues. The positive residues, the charge cluster, hydrophobic core, palindrome and the conserved 100–104 domain are highlighted. Arrow: α-cleavage site. **B**: Western blot of PNGase treated cell lysates of Hpl cells transfected with the PrP^C^ deletion constructs used in the current study. Detection was done with POM1. Arrows point to the full length PrP (FL) and C1 fragment. **C**: Quantification of the percentage of α-cleavage of the various PrP^C^ mutants, based on densitometry of western blots, from which (B) is representative. Quantifications were calculated in the linear range of the densitometric signal of independent experiments. Values refer to the amount of C1 generation comparing to total abundance of PrP^C^, and are normalized to cleavage of PrP^C^, which was assessed in the same blot. Error bars represent the SEM. **D**: Graphic illustrating the percentage of α-cleavage impairment of PrP^C^ deletion mutants. Points correspond to the average value illustrated in (C), for the samples PrP^C^, BOM16, BOM15, BOM17 and C40, which have 0, 4, 9, 14 and 30 amino acids deleted, respectively. In x-axis are plotted the size of the deletion of each of these constructs. Dashed line refers to the estimated deletion size that would result in 50% inhibition of α-cleavage.

Because PrP^C^ proteolysis was not prevented by the point mutations enumerated above, whereas larger deletions within the HC impaired α-cleavage, we extended our investigations of PrP^C^ deletions. We firstly assessed the cleavage efficiency of C40, the construct with a 30-residue deletion encompassing most CC and HC (Fig. 4A). Essentially no C1 was observed when the C40 construct was transfected into Hpl cells (Fig. 4B–C), thereby establishing that the region encompassing residues 100–129 is essential for modulating α-cleavage of PrP^C^. To define the minimal region essential for α-cleavage, the generation of C1 was assessed in PrP^C^ constructs bearing smaller deletions within the stretch 100–129. BOM12 and BOM13 had a deletion of the α-cleavage site and of further upstream residues (Fig. 4A), whereas C39 contained a deletion of the entire palindrome downstream of the α-cleavage site. Finally, BOM14 had a deletion downstream of the palindrome but maintained the α-cleavage site (Fig. 4A).

No differences in α-cleavage were detected between BOM12, BOM14, and PrP^C^ (Fig. 4B–C). In contrast, BOM13, with deletion in the residues 105–110, and C39, which lacks the domain 111–120, showed about 50% reduction in C1 abundance (Fig. 4B–C). This suggests that the space occupied by residues 105–120, but not necessarily the chemical identity of those residues (Fig. 2), is important for α-cleavage. Surprisingly, although the removal of the region 100–110 did not affect α-cleavage in BOM12, the shorter deletion of 105–110 resulted in about 50% inhibition of α-cleavage in BOM13. This suggests a negative modulation of cleavage by residues 100–104 in this context. The sequence present in BOM13, but absent from BOM12, is KPSKP (Fig. 4A) and is highly conserved domain in mammals [32], [33]. Even lower vertebrates, including chicken and fish, generally conserve the KPxKP motif [33], [34]. Perhaps the α-PrPase is partially blocked in BOM13, but not in BOM12, due to an entity interacting with the conserved region 101–104. Alternatively, this constellation of prolines and charged residues may contribute to a decrease in the flexibility of this segment and, in turn, enhance the antagonistic effects of the deletion on the efficiency of α-cleavage.

Because the domain 105–120 appeared to be important for α-cleavage, we set out to identify the minimal region within this domain that would affect the proteolytic processing of PrP^C^. For this purpose, we assessed the degree of C1 generation in the mutants BOM17, BOM15, and BOM16 bearing deletions of 14, 9, and 4 residues within the domain 105–120, respectively (Fig. 4A). Quantification of PrP^C^ proteolysis in these constructs revealed a C1 reduction by about 28% for BOM16 (4-residue deletion; Fig. 4B–C). The extent of cleavage impairment was proportional to the size of the deletions (Fig. 4B–D), provided that said deletions encompassed the α-cleavage site and were situated within the domain 106–119. When the domain 106–119 was deleted, α-cleavage was only marginally higher than in C40 (Fig. 4B–D). These findings suggest that α-cleavage is not controlled by a specific primary sequence, but rather by the length of the unstructured stretch encompassing residues 105–120. Accordingly, 50% cleavage inhibition is expected to be achieved with a deletion of six residues (Fig. 4D).

### α-Cleavage of Neurotoxic PrP Variants

**Figure 5:**
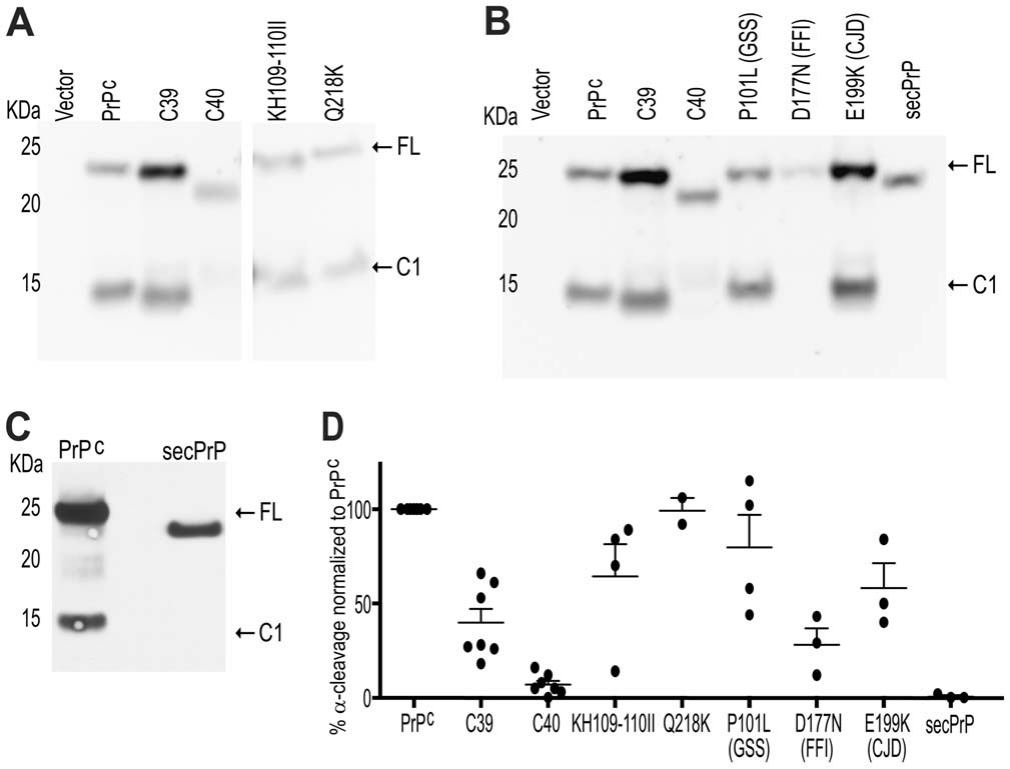
α-cleavage of PrP^C^ mutants with PrP^Sc^-generating point mutations. **A–B**: Western blots of PNGase treated cell lysates of Hpl cells transfected with various PrP mutants. All lanes in (A) belong to the same blot. Detection was done with POM1. Arrows point to the full length PrP (FL) and C1 fragment. **C**: Western blot of PNGase treated brain homogenates of mice expressing GPI-anchorless secreted PrP^C^ in *Prnp^−/−^* background, and wild-type PrP^C^. **D**: Quantification of the percentage of α-cleavage of the various PrP^C^ mutants, based on densitometry of western blots, from which (A) and (B) are representative. Quantifications were calculated in the linear range of the densitometric signal of independent experiments. Values refer to the amount of C1 generation comparing to total abundance of PrP^C^, and are normalized to cleavage of wild-type PrP^C^ which was assessed in the same blot. Error bars represent the SEM.

Increased hydrophobicity in the HC may result in increased generation of a transmembrane species of PrP termed ^Ctm^PrP, which may be toxic [35]. This was mainly supported by a study where toxicity and prion replication were assessed in a transgenic mouse model that highly overexpressed the mutant PrP^KH109–110II^ in the absence of wild-type PrP^C^ [35]. This PrP^C^ mutant had the two charged amino acids of the α-cleavage site substituted by hydrophobic isoleucines. It was suggested that enhanced hydrophobicity on the PrP molecule led to higher ^Ctm^PrP formation and increased PrP^Sc^ accumulation [35]. Here we assessed whether the toxicity of this mutant might be explained by inhibition of α-cleavage. For this purpose the proteolysis of PrP^KH109–110II^ was assessed in Hpl cells. Quantification of α-cleavage suggested that this may be partially inhibited (Fig. 5A,D), yet the yield of C1 was very variable and ranged between 11% and 30% inhibition. In one single sample we observed 86% inhibition.

In order to further test the hypothesis that a slight impairment on PrP^C^ cleavage could be mildly toxic and therefore be translated into human diseases, the degree of proteolysis of constructs carrying mutations linked to human prion diseases was quantified. PrP^P101L^ is associated with Gerstmann-Sträussler-Scheinker (GSS) syndrome [36], PrP^D177L^ replicates fatal familial insomnia (FFI) [37], and PrP^E199K^ is found in familiar CJD cases [38], [39]. For control, the proteolysis of PrP^Q218K^ was assessed, which is a construct reported to be a dominant negative of PrP^Sc^ replication [40]. Another control was secPrP^C^, which is secreted and has been shown to be convertible into PrP^Sc^, but not fatal in mice [41]. Quantification of C1 generation in Hpl cells expressing these mutants showed reduced α-cleavage in PrP^E199K^, and even more so in PrP^D177N^ (Fig. 5B,D). Also, PrP^P101L^ showed a high variability in terms of α-cleavage rate, with low values of C1 generation observed sporadically (Fig. 5B,D). In contrast, the PrP^Sc^ dominant negative PrP^Q218K^ was cleaved in a similar fashion as normal PrP^C^ (Fig. 5A,D), suggesting a link between toxicity of inherited human prion diseases, and inhibition of α-cleavage of PrP. However, secPrP^C^ did not show any C1 generation in Hpl cell lysates (Fig. 5B,D), in contrast to a previous report using human SH-SY5Y cells [13]. In order to clarify this discrepancy, we assessed the cleavage of this mutant in brain of transgenic mice expressing secPrP^C^ in *Prnp^−/−^* mice [41]. Also here we were not able to detect any C1 fragment (Fig. 5C). We conclude that secPrP^C^ does not generate cell-associated C1 in the systems analyzed here. This finding, in combination with results reported elsewhere [42], suggests that the GPI anchor is necessary for the toxicity of uncleavable PrP^C^ mutants.

## Discussion

α-cleavage of PrP^C^ occurs in many cell types and tissues at a well-defined site located in the unstructured portion of the protein [10], generating a stable carboxy terminal fragment of ca. 15 kDa [7], [14], [43] and an N-terminal fragment of ca. 12 kDa [27], [28]. The robust and stereotypic nature of these processing events suggests the existence of specific proteases which we term PrPases. However, we were surprised to find that PrP^C^ proteolysis is remarkably tolerant of variations in the sequence surrounding the site of cleavage. A large number of manipulations had little or no effect in α-cleavage of PrP^C^; some of these manipulations were very far-reaching and included removal of the canonical cleavage site, inversion of all charges, radical manipulations of the hydrophobicity, and alteration of the palindrome domain.

As the resolution of SDS-PAGE does not allow for detecting single amino-acid shifts in the cleavage site, it is possible that some of the PrP^C^ mutants may have experienced subtle shifts in the α-cleavage site. Even if that were the case, all manipulations consistently preserved the generation of a C1 cleavage product whose abundance and electrophoretic motility was similar to bona fide C1 generated by proteolysis of wild-type PrP^C^. Conversely, deletion of residues 106–119 strongly reduced α-cleavage, whereas shorter deletions within this region had only a moderate effect. The efficiency of cleavage was inversely proportional to the size of deletions centered onto the α-cleavage site.

The results described in this study allow drawing several surprising conclusions on the molecular preconditions for α-cleavage of PrP^C^. Firstly, substitution of positively charged residues at the α-cleavage site has no major effect on the degree of PrP^C^ proteolysis. This is in agreement with previous studies of N2a cells transfected with GFP-fused ovine PrP^C^ mutants [21]. Secondly, replacement of all six positive charges of the CC with negative charges, including those of the α-cleavage site, resulted only in a partial inhibition of C1 generation. Thirdly, even total or partial substitution of the palindromic alanines flanking the α-cleavage site with glycines had little impact in the normal proteolysis of PrP^C^. This observation contradicts a previous report in murine N2a cells [19], yet was particularly robust in our experience and appears to be valid in both murine and human cells. Fourthly, amino acid substitutions reducing the hydrophobicity of the region surrounding the α-cleavage site failed to reduce the proteolysis of PrP^C^. However, impairment of α-cleavage was observed upon substitution of the charged residues within the cleavage site for two highly hydrophobic isoleucines. Fifthly, deletions in the vicinity of the α-cleavage site, as the domains 100–110 and 121–129, had no impact onto cleavage whereas deletions of the domains 105–110 and 111–120 resulted in about 50% impairment on α-cleavage. The latter result is in agreement with our previous report that a PrP^C^ mutant lacking residues 114–121 undergoes inefficient α-cleavage in transgenic mice [14].

It is somewhat counterintuitive that deletion of residues 100–110 had no apparent effect whereas the shorter deletion 105–110 partially blocked α-cleavage. This finding points to the importance of the 100–104 KPSKP residues. This region is highly conserved in vertebrates [33], [34] and has been suggested to be important for protein binding [44]. One of the domains indentified in the consensus sequence in vertebrates is PxxP, which is an SH3-binding motif [34], [44], [45]. All of the above suggests the existence of a macromolecule that interacts with the domain 100–104 and may occlude the α-cleavage site. This putative macromolecule may not necessarily be a protein. Alternatively, a proline kink at the α-cleavage site may impair the function of the α-PrPase in *cis*.

In our experiments, the C1 fragment was suppressed below detectability only when the entire segment 106–119, or major portions thereof, was removed. However, shorter deletions within the domain 106–119 centered in the α-cleavage site region only partially inhibited proteolysis of PrP^C^. Assuming that the efficiency of cleavage is linearly dependent on the length of this segment, a 50% blockage of α-cleavage would be obtained with a six-residue deletion.

In summary, α-cleavage of PrP^C^ has a strong tolerance towards sequence degeneration. Such tolerance may underlie evolutionary pressure, since PrP^C^ mutants that do not undergo α-cleavage are neurotoxic [14], [15], [16]. We found the determinants of PrP^C^ cleavage to be invariant in many biological systems, including Npl cells derived from *Prnp^−/−^* mice [24], human HeLa cells, mouse embryonic fibroblasts, and transgenic mice [14]. This suggests that the relevant α-PrPases are functionally conserved and present in many tissues. What might underlie the surprising resilience of α-cleavage to sequence variation? Maybe several distinct proteases display redundant α-PrPase activity. If that were the case, alterations of the cleavage site may affect the recognition by some–but not all–proteases, resulting in unhindered global α-PrPase activity. Alternatively, PrP^C^ may be processed by a single α-PrPase with high plasticity of its substrate specificity. Examples of such proteases are the desintegrin and metalloproteases (ADAM) 10 and 17 (also known as TACE, or α-secretase of APP). These proteins have been reported to cleave a broad range of basic, acidic, or zwitterionic substrates [46] with no common consensus sequences [46], [47], [48], and have therefore been also termed “sheddases”. The activity of sheddases is typically tolerant of point mutations [49], [50] and their cleavage sites appears to rely on the distance of unfolded substrate regions from the cell membrane [49]. Analogously, the α-cleavage site of PrP^C^ is localized in an unfolded region and is flanked by a hydrophobic domain which may associate with the cell membrane [31]. Analogously to various substrates of ADAM 10 and 17, the cleavage of PrP^C^ appears to tolerate charge removal or inversion. Indeed, ADAM-10 and ADAM-17 have been suggested to act as α-PrPases [51], [52], yet this assertion is not entirely uncontroversial. The function of ADAMs necessitates divalent cations, and it was reported that 10 µM of EDTA does not inhibit α-cleavage [12], yet others have reported that 5 mM EDTA inhibits cleavage in a Cu^2+^ and Fe^2+^ dependent manner [43].

Some of the mutations analogous to those found in familial prion diseases appear to impair the proteolytic processing of PrP^C^. In addition, α-cleavage appeared to be also impaired in PrP^KH109–110II^, which is toxic in transgenic mice [35]. In construct PrP^Q218K^, which dominantly inhibits PrP^Sc^ replication [40], the degree of α-cleavage was similar to the one detectable in PrP^C^. It remains to be established whether PrP^C^ cleavage inhibition is a cause or a consequence of the toxicity of these mutants. In this context it may be interesting to assess the degree of cleavage of these constructs in transgenic mice before the onset of the disease.

Through the study of a large panel of transgenic mice expressing many different PrP^C^ mutants, we found that the toxicity of PrP^C^ deletion mutants was abolished by removal of the GPI anchor [42]. This finding argues that toxicity is exerted through some process that occurs at the membrane, such as interaction with signal-transducing molecules. In this context it is interesting to note that infection of transgenic mice expressing secPrP, which did not produce any C1 fragment in their brains, results in only weak neurotoxicity [41]. These results may be taken to suggest that similar neuropathogenic cascades are activated both in scrapie infection and in expression of non-cleavable PrP^C^ variants.

Absence of α-cleavage is characteristic shared by PrP^Sc^ [7], [43] and toxic PrP^C^ mutants [14], [15], [16], suggesting that α-cleavage is important for the function of PrP^C^ and possibly even for the pathogenesis of prion diseases. Yet the identity and the functional characteristics of the enzymes relevant for this process are still unknown. A promising way forward may take advantage of newly developed antibodies [26] that allow for isolation and native elution of PrP^C^-containing supramolecular assemblies [53]. The differential analysis of multiprotein complexes containing variants of PrP^C^ that are permissive or refractory to cleavage may lead the identification of the relevant sheddases and possibly also of downstream players involved in prion toxicity.

## Materials and Methods

### Cloning

Murine PrP^C^ was amplified from total brain cDNA, using the primers SY6 and SY7 (Table S1), which introduces the *Bam*HI and the *Sal*I cleavage sites, respectively. The PCR products were digested with *Bam*HI and *Sal*I and the targeted vector, pBMN-I-EGFP (Addgene, plasmid 1736), was digested with *Bam*HI and *Xho*I. Fragments were later purified from an agarose gel, using the Amersham GFX PCR DNA and Gel Band Purification Kit. The PCR products and the open plasmid were ligated using the Roche Rapid DNA Ligation Kit. The products were once again purified using Amersham GFX PCR DNA and Gel Band Purification Kit, and transformed into TOP10 competent *E. coli* (Invitrogen). Other constructs were performed in two PCR steps using the primers and templates listed in Table S1. In the first step, PCR was performed with: 1) SY6 (Table S1) and the reverse plasmid referred in Table S1; 2) SY7 (Table S1) and the forward plasmid referred in Table S1. The second step was performed with a PCR using 1 uL of PCR product 1) and 2), and using SY6 and SY7 to make the full construct. The construct secPrP was cloned by one additional step with SY6 and A33 primers (Table S1). PCR product purification, digestion and ligation were done in a similar way as described for PrP^C^. Constructs PrP^Δ118–125^ and PrP^Δ111–125^ were made using the QuikChange kit (Stratagene), using the primers indicated in Table S1. Dv1 construct originates from previous experiments [54].

### Analysis of the Constructs

Most of the work was performed in Hpl cells [25]. Other cell lines used were N2a-Pk1 [22], NIH-3T3 [23], Npl [24], Hela [29]. Primary embryonic fibroblasts obtained from E12,5 *Prnp^−/−^* embryos and brains from mice were also used.

Transfection was performed using Lipofectamine (Invitrogen) and Plus Reagent (Invitrogen), into 80% confluent cells. Cells were harvested using 10 mM EDTA, 2 days after transfection. Cell lysis was performed on ice for 1 h, using 1% Triton-X-100 and 1% NP-40, in Tris buffer at pH 7.5, with NaCl and protease inhibitors (Complete; Roche). For brain lysis, a 10% homogenate was prepared in Ripa buffer with protease inhibitors (Complete; Roche), and lysed for 1 h on ice. For all samples, lysate supernatant was collected after centrifugation for 30 min at 16000 g at 4°C. Supernatant was then treated with PNGase (NEB), and loaded onto 12% SDS-polyacrylamide gels. After electrophoresis, proteins were transferred to nitrocellulose membranes (Schleicher & Schuell) by wet blotting. Primary antibody was anti PrP^C^ POM1 [26], POM11 [26], or POM19 [26] in case of human derived Hela cells, diluted 1∶10'000 from a 1 ug/ul stock. Peroxidase-labelled anti-mouse IgG1 diluted 1∶10'000 (Zymed) was used as secondary antibody. Antibodies were probed for 1 h at room temperature or over-night at 4°C in a 1% Top-Block solution (FLUKA) in PBS-Tween 20. For membrane blocking, a 5% Top-Block solution was used. Development was done using ECL detection system (Pierce). Densitometric assessment of PrP bands was performed using TINA v2.09g (Raytest Isotopenmessgeräte), in the linear range of the band intensity. Hydrophobicity plots were calculated for using DNAMAN software (Lynnon BioSoft, Canada), for window intervals of nine residues.

### Immunoprecipitation and Western Blot Analysis of N-Fragment

For each sample, immunoprecipitation was performed in 7 mL of Optimem media, supplemented with a protease inhibitors (Complete; Roche), and collected after 8 h culture of 90% confluent cells in T75 flasks. Dynabeads M-280 tosyl-activated (Invitrogen) were coated according to manufacture procedures. Beads were washed twice with 0.5% CHAPS, 0.5% NP-40, and twice with 1% CHAPS, 1% NP-40. Elution was performed by boiling the beads for 10 min at 95°C in Loading Buffer 2X (Invitrogen).

## Supporting Information

Table S1 List of the primers used in this study.
(0.11 MB DOC)
