# Supplementary material for: Unexpected Tolerance of α-Cleavage of the Prion Protein to Sequence Variations
Source: PLoS One. 2010 Feb 8;5(2):e9107. doi: 10.1371/journal.pone.0009107 (PMC2817006; doi:10.1371/journal.pone.0009107)
Supplement: Table S1 — List of the primers used in this study. (0.11 MB DOC) [file pone.0009107.s001.doc]

## Supplementary Tables

Table S1: List of the primers used in this study.

| Primer name | sequence 5' ---> 3' | direction | Description | Code | Template |
| --- | --- | --- | --- | --- | --- |
| A5 | aaa cca aaa acc aac ctc gat gat gtg gca ggg gct gcg gca | Fw | PrP (KH109-110DD) | BOM3 | PrP^C^ |
| A6 | tgc cgc agc ccc tgc cac atc atc gag gtt ggt ttt tgg ttt | Rv | PrP (KH109-110DD) | BOM3 | PrP^C^ |
| A11 | aaa cca aaa acc aac ctc gcc gcc gtg gca ggg gct gcg gca | Fw | PrP (KH109-110AA) | BOM6 | PrP^C^ |
| A12 | tgc cgc agc ccc tgc cac ggc ggc gag gtt ggt ttt tgg ttt | Rv | PrP (KH109-110AA) | BOM6 | PrP^C^ |
| A13 | aaa cca aaa acc aac ctc gtg gca aag cat ggg gct gcg gca | Fw | PrP (KHVA109-112VAKH) | BOM7 | PrP^C^ |
| A14 | tgc cgc agc ccc atg ctt tgc cac gag gtt ggt ttt tgg ttt | Rv | PrP (KHVA109-112VAKH) | BOM7 | PrP^C^ |
| A15 | ggg ggt acc cat aat cag tgg aac gcc ccc agc gca cca gca acc aac ctc gcc gcc gtg gca ggg gc | Fw | PrP (K100,103,105,109A H110A) | BOM8 | BOM6 |
| A16 | gc ccc tgc cac ggc ggc gag gtt ggt tgc tgg tgc gct ggg ggc gtt cca ctg att atg ggt acc ccc | Rv | PrP (K100,103,105,109A H110A) | BOM8 | BOM6 |
| A17 | aaa acc aac ctc aag cat gtg gga ggg ggt ggg gga ggt ggg gga gta gtg ggg ggc ctt ggt | Fw | PrP (A112G,A114-117G,A119G) | BOM9 | PrP^C^ |
| A18 | acc aag gcc ccc cac tac tcc ccc acc tcc ccc acc ccc tcc cac atg ctt gag gtt ggt ttt | Rv | PrP (A112G,A114-117G,A119G) | BOM9 | PrP^C^ |
| A21 | aaa acc aac ctc aag cat gtg gga ggg gct ggg gca ggt ggg gca gta gtg ggg ggc ctt | Fw | PrP (A112,115,117G) | BOM11 | PrPc |
| A22 | aag gcc ccc cac tac tgc ccc acc tgc ccc agc ccc tcc cac atg ctt gag gtt ggt ttt | Rv | PrP (A112,115,117G) | BOM11 | PrP^C^ |
| A23 | gtg gca ggg gct gcg gca gct | Fw | PrP Δ(100-110) | BOM12 | PrP^C^ |
| A24 | ccc tgc cac gtt cca ctg att atg ggt acc ccc t | Rv | PrP Δ(100-110) | BOM12 | PrP^C^ |
| A23 | gtg gca ggg gct gcg gca gct | Fw | PrP Δ(105-110) | BOM13 | PrP^C^ |
| A26 | ccc tgc cac tgg ttt gct ggg ctt gtt cca ctg att | Rv | PrP Δ(105-110) | BOM13 | PrP^C^ |
| A27 | ggg agc gcc atg agc agg ccc at | Fw | PrP Δ(121-129) | BOM14 | PrP^C^ |
| A28 | ggc gct ccc tac tgc ccc agc tgc cgc agc ccc t | Rv | PrP Δ(121-129) | BOM14 | PrP^C^ |
| A29 | gc aaa cca aaa gca gct ggg gca gta gtg ggg ggc ctt | Fw | PrP Δ(106-114) | BOM15 | PrP^C^ |
| A30 | ttt tgg ttt gct ggg ctt gtt | Rv | PrP Δ(106-114) | BOM15 | PrP^C^ |
| A31b | aa acc aac ctc gct gcg gca gct ggg gca gta | Fw | PrP Δ(109-112) | BOM16 | PrP^C^ |
| A32 | gag gtt ggt ttt tgg ttt gct | Rv | PrP Δ(109-112) | BOM16 | PrP^C^ |
| A34 | gc aaa cca aaa gta gtg ggg ggc ctt ggt ggc ta | Fw | PrP Δ(106-119) | BOM17 | PrP^C^ |
| A30 | ttt tgg ttt gct ggg ctt gtt | Rv | PrP Δ(106-119) | BOM17 | PrP^C^ |
| A33 | gtc gac cct ccc tca gga tct tct ccc gtc gta ata ggc ctg | Rv | secPrP | BOM18 | PrP^C^ |
| B14 | aaa acc aac ctc aag cat ¦ ggc tac atg ctg ggg agc | Fw | PrP Δ(111-125) | BOM23 | PrP^C^ |
| B15 | gct ccc cag cat gta gcc ¦ atg ctt gag gtt ggt ttt | Rv | PrP Δ(111-125) | BOM23 | PrP^C^ |
| B16 | gca ggg gct gcg gca gct ¦ ggc tac atg ctg ggg agc | Fw | PrP Δ(118-125) | BOM24 | PrP^C^ |
| B17 | gct ccc cag cat gta gcc ¦ agc tgc cgc agc ccc tgc | Rv | PrP Δ(118-125) | BOM24 | PrP^C^ |
| B18 | aag cat gtg gca ggg gct ggg gga gct ggg gca caa cag ggg ggc cag ggt ggc tac atg cag ggg agc gcc atg agc agg | Fw | PrP (A115-116G, V120-121Q, L124Q, L129Q) | BOM25 | PrP^C^ |
| B19 | cct gct cat ggc gct ccc ctg cat gta gcc acc ctg gcc ccc ctg ttg tgc ccc agc tcc ccc agc ccc tgc cac atg ctt | Rv | PrP (A115-116G, V120-121Q, L124Q, L129Q) | BOM25 | PrP^C^ |
| B20 | aag cat gtg gca ggg gct ggg gga gct ggg gca gta gtg ggg ggc cag ggt ggc tac atg cag ggg agc gcc atg agc agg | Fw | PrP (A115-116G, L124Q, L129Q) | BOM26 | PrP^C^ |
| B21 | cct gct cat ggc gct ccc ctg cat gta gcc acc ctg gcc ccc cac tac tgc ccc agc tcc ccc agc ccc tgc cac atg ctt | Rv | PrP (A115-116G, L124Q, L129Q) | BOM26 | PrP^C^ |
| B22 | gct gcg gca gct ggg gca caa cag ggg ggc cag ggt ggc tac atg cag ggg agc gcc atg agc agg | Fw | PrP (V120-121Q, L124Q, L129Q) | BOM27 | PrP^C^ |
| B23 | cct gct cat ggc gct ccc ctg cat gta gcc acc ctg gcc ccc ctg ttg tgc ccc agc tgc cgc agc | Rv | PrP (V120-121Q, L124Q, L129Q) | BOM27 | PrP^C^ |
| BAUF95 | t cag tgg aac gag ccc agc gaa cca gaa acc aac ctc gag cat gtg gc | Fw | PrP (K100,103,105,109E) | FBOM1 | PrP^C^ |
| BAUF96 | gc cac atg ctc gag gtt ggt ttc tgg ttc gct ggg ctc gtt cca ctg a | Rv | PrP (K100,103,105,109E) | FBOM1 | PrP^C^ |
| BAUF97 | ga ggg ggt acc gag aat cag tgg aac gag ccc agc gaa cca aaa ac | Fw | PrP (H95E, K100E, K103E) | FBOM2 | PrP^C^ |
| BAUF98 | gt ttt tgg ttc gct ggg ctc gtt cca ctg att ctc ggt acc ccc tc | Rv | PrP (H95E, K100E, K103E) | FBOM2 | PrP^C^ |
| BAUF99 | ccc agc gaa cca gaa acc aac ctc gag gag gtg gca ggg g | Fw | PrP (H95E, K100E, K103E, K105E, K109E, H110E) | FBOM3 | FBOM2 |
| BAUF100 | c ccc tgc cac ctc ctc gag gtt ggt ttc tgg ttc gct ggg | Rv | PrP (H95E, K100E, K103E, K105E, K109E, H110E) | FBOM3 | FBOM2 |
| DOM2f | gtg cgt cac cca gta caa gaa gga gtc cca ggc | Fw | PrP (Q218K) | Dv1 | PrP^C^ |
| DOM2r | gcc tgg gac tcc ttc ttg tac tgg gtg acg cac | Rv | PrP (Q218K) | Dv1 | PrP^C^ |
| Bauf5 | gca aac caa aaa cca acc tca tca tcg tgg cag ggg ctg cgg cag | Fw | PrP (KH109-110II) | C12 | PrP^C^ |
| Bauf6 | ctg ccg cag ccc ctg cca cga tga tga ggt tgg ttt ttg gtt tgc | Rv | PrP (KH109-110II) | C12 | PrP^C^ |
| SY71 | cag tgg aac aag ctc agc aaa cca aaa | Fw | PrP (P101L) | C32 | PrP^C^ |
| SY72 | ttt tgg ttt gct gag ctt gtt cca ctg | Rv | PrP (P101L) | C32 | PrP^C^ |
| SY73 | aac ttc gtg cac aac tgc gtc aat atc | Fw | PrP (D177N) | C33 | PrP^C^ |
| SY74 | gat att gac gca gtt gtg cac gaa gtt | Rv | PrP (D177N) | C33 | PrP^C^ |
| SY75 | gag aac ttc acc aag acc gat gtg aag | Fw | PrP (E199K) | C34 | PrP^C^ |
| SY76 | ctt cac atc ggt ctt ggt gaa gtt ctc | Rv | PrP (E199K) | C34 | PrP^C^ |
| SY83 | ctc aag cat gtg ggg ggc ctt ggt | Fw | PrP Δ(111-120) | C39 | PrP^C^ |
| SY82 | gcc ccc cac atg ctt gag gtt ggt | Rv | PrP Δ(111-120) | C39 | PrP^C^ |
| SY85 | cag tgg aac ggg agc gcc atg agc | Fw | PrP Δ(100-129) | C40 | PrP^C^ |
| SY84 | ggc gct ccc gtt cca ctg att atg | Rv | PrP Δ(100-129) | C40 | PrP^C^ |
| SY6 | cgc gga tcc aat tta gga gag cca agc aga | Fw | PrP^C^ |  |  |
| SY7 | acg cgt cga cca cga gaa tgc gaa gga aca | Rv | PrP^C^ |  |  |
